# Recommendations for improving accessibility of digital health interventions for cardiometabolic disease for ethnically diverse populations

**DOI:** 10.1177/20552076241272600

**Published:** 2025-01-28

**Authors:** Mel Ramasawmy, Nushrat Khan, David Sunkersing, Madiha Sajid, Shivali H Modha, Manoj Mistry, Wasim Hanif, Fiona Stevenson, Kiran Patel, Kamlesh Khunti, Paramjit Gill, Lydia Poole, Amitava Banerjee

**Affiliations:** 1 Institute of Health Informatics, UCL, London, UK; 2 Wolfson Institute of Population Health, Queen Mary University of London, London, UK; 3 Department of Primary Care and Public Health, Imperial College London, London, UK; 4 DISC Study, Patient and Public Involvement Representative, London, UK; 5 University Hospitals Birmingham, Birmingham, UK; 6 Department of Primary Care and Population Health, University College London, London, UK; 7 Warwick Medical School, University of Warwick, Coventry, UK; 8 Diabetes Research Centre, Leicester General Hospital, University of Leicester, Leicester, UK; 9 Department of Psychological Interventions, School of Psychology, University of Surrey, Guildford, Surrey, UK; 10 Department of Cardiology, Barts Health NHS Trust, London, UK

**Keywords:** Cardiovascular disease, diabetes, digital health, eHealth, qualitative

## Abstract

Digital health interventions (DHIs), such as apps, websites and wearables, are being presented as solutions or enablers to manage the burden of cardiometabolic disease in healthcare. However, the potential benefits of DHIs may not be reaching the most in-need populations, who may face intersecting barriers to accessing health services and digital solutions. The Digital Interventions for South Asians in Cardiometabolic Disease (DISC) study used a mixed-method approach to focus on people of a South Asian background, a high-risk group for cardiometabolic disease. A one-day workshop was held in May 2023 with key stakeholders, including people with lived experience, health professionals, technology innovators and policymakers (*n* = 34), to develop recommendations arising from the DISC study findings. Discussions covered four areas: actions to support individuals to access and benefit from DHIs; translating learning about inclusive design into practice; the role of regulation and evaluation to improve inclusivity of DHIs used within the health service; and improving quality of data collection and use to reduce inequalities related to digital health and cardiometabolic disease. Our recommendations align with recent national strategies and provide specific examples of actions that can be taken to address digital inequalities for ethnic minority populations.

## Background: Findings of the Digital Interventions for South Asians in Cardiometabolic Disease (DISC) study

Digital health interventions (DHIs) are of growing interest in health policy, accelerated by the adoption of digital approaches in health and care services during the COVID-19 pandemic.^1,2^ Cardiovascular disease and diabetes mellitus (together referred to as ‘cardiometabolic disease’) represent the largest causes of burden of disease in the UK and globally.^3,4^ Potential benefits of DHI use in cardiometabolic disease include improved patient knowledge and outcomes, and healthcare cost savings,^5–8^ although evidence of long-term effectiveness is lacking. However, digital approaches may exacerbate existing inequities through intersecting factors influencing digital exclusion and social determinants of health.^9^ The DISC study looked at the use of DHIs in people of South Asian background with heart disease and diabetes, as this group are at a higher risk of these conditions,^10^ and some communities may face more barriers to using digital health.^11^ Understanding facilitators and barriers to DHI uptake, use and effectiveness in this population could help improve equitable approaches to digital health care more generally.

Through our scoping review of frameworks for implementation, uptake, and use of cardiometabolic disease-related DHIs in ethnic minority populations,^12^ we developed a guide of the key constructs across the four potential levels of action for digital health inequalities. We used this guide to support our analysis of facilitators and barriers to DHI uptake and use at individual,^13,14^ healthcare professional,^15^ as well as systems and policy levels.^16^ Many South Asian individuals were interested in DHIs if they aided self-management of their health, but described barriers for themselves and others, including the cost of DHIs, devices and internet access; skills or confidence in using digital technology; digital literacy (the ability to find, evaluate, and communicate information safely online); options for non-English languages, and awareness of available DHIs. Health and care staff reported they needed more information and training to confidently recommend DHIs to patients and often did not have time to introduce and support patients for DHIs during consultations. While there was good awareness among technology innovators of how DHIs can be designed to be useful to more of the population, there is a need for improved evaluation, shared learning, and regulation to ensure equitable benefit. Participants working at a national policy and strategy level highlighted that ensuring equitable access to digital health is complex and is often exacerbated by broader sociodemographic inequalities, lack of integration within the National Health Service (NHS), and lack of shared understanding of required actions to tackle these inequalities.

## DISC study recommendations workshop

In May 2023, the DISC study team brought together key stakeholders, for a full-day workshop to develop policy recommendations addressing inequalities related to digital health and cardiometabolic disease. In total, there were 34 attendees online and in person (in the following capacities: people with lived experience/patient and public involvement representatives (*n* = 10), NHS and clinical (*n* = 4), DHI companies (*n* = 2), policy and strategy (*n* = 5), academic, clinical academic or research commissioning and support (*n* = 7), and charity or community organisations (*n* = 6)). Four key topics were identified based on the DISC study findings: enabling patient uptake and use; DHI design; DHI evaluation and regulation; and data related to inequalities. Findings from the DISC study were presented, and each topic was introduced with an expert-led presentation, including from South Asian people living with cardiometabolic disease. Participants were split into small groups of 4–6 and asked to develop and submit recommendations around each topic, which were collated by the study team. Finally, participants independently prioritised recommendations using an anonymous online voting system, Mentimeter. Attendees were informed that the outcomes of the workshop would be published and were included in the acknowledgements if they provided consent by email.

## Recommendations

The final list of recommendations included practical solutions, such as offering free or low-cost devices, as well as system-level changes to regulation of digital health to embed equitable approaches. While the DISC study focused on experiences of people of South Asian background in the UK, many recommendations had relevance across groups at risk of digital exclusion, as well as for the overall improvement of the quality of DHIs available through the NHS. A full list of recommendations can be found in Supplemental 1 in the online supplemental material. Here, we summarise the outputs from these discussions.

### What actions can we take to reduce barriers to access to and benefits from, digital health for cardiometabolic disease?

The discussion emphasised the importance of ensuring that digital forms of engagement were not the only choice for everyone accessing the health system. However, to support those who may benefit from DHIs, recommendations focused on engaging communities affected by the health condition of interest in the entire process, including working with communities to build trust in digital health and understand their interests and needs. Opportunities to digitally enable people included offering digital devices to those who need them, as well as providing training and support via community groups, ‘digital occupational therapists’, and signposting from primary care to online and media resources. Taking these steps could support more diverse groups to engage in DHI design, as well as improve acceptability during implementation.

### How can we appropriately embed learning about inclusive design and user testing in the development of digital health tools for cardiometabolic disease?

A key question in discussions about user-centred design was how DHI creators can learn more about patient needs and preferences when developing an intervention, requiring more efforts to improve the accessibility of the design process to a wider range of people. It was recognised that it can be difficult to balance meaningful patient engagement with the speed and agility of the design process. However, it was considered important to do so. Suggestions included involving the user as a ‘partner’ rather than the ‘subject’, through early engagement with community groups in the ideation or design stage, and recognising input through compensation for their time. Those involved in development of DHIs also spoke about the need for guidance on improving accessibility for different groups in design of the end product, building on existing guidance relating to vision, motor, hearing and cognitive impairments; and opportunities to share learning and good practice between projects that have successfully designed and implemented DHIs in diverse populations.

### How can regulation and evaluation improve inclusivity of digital health tools for heart disease and diabetes?

Recommendations in this area emphasised the need to improve evidence and evaluation of DHIs, including development of frameworks for evaluation for DHIs that address whether an intervention works for different user groups in the long term, compared to usual care. This was further developed into a recommendation for evidence-based standards for DHIs in accreditation for NHS use, with specific advice and examples and case studies to support developers. Clear leadership, governance and accountability were also highlighted, including ensuring that funders and commissioners of DHI research or services understand ‘what good looks like’, and consequences of failing to meet standards.

### How do we improve data quality and access to understand and reduce inequalities related to digital health and cardiometabolic disease?

The first theme that emerged in this area focused on building public trust by ensuring an understanding of the benefits, risks, and rights in relation to data collection. The second theme focussed on improving data quality to improve how we understand and address health inequalities. Suggestions included: training and support for staff to help collect potentially sensitive data; improving interfaces for inputting data; communicating the limitations of the data for those conducting or using analyses; and regular review of categories for data that address health inequalities to ensure they are meaningful and appropriate to address needs. For example, to improve service delivery to a particular ethnic group, an NHS Trust or local authority may need data at a higher level of granularity than the ONS 18 categories.^17^ The third theme in this discussion focused more broadly on questions around trust, and the need to clarify obligations of industry with respect to health data (particularly if commissioned by the NHS).

## Discussion

In the past decade, UK policymakers have paid significant attention to digital health, including through the Department of Health and Social Care Medical Technology Strategy,^18^ and NHS-commissioned research on improving motivation for the uptake of digital services.^19^ Previous studies have made recommendations on how to increase DHI uptake in cardiometabolic disease^20^; and highlighted the lack of patient-centred or co-designed approaches to DHIs in cardiovascular care.^21^ The NHS Race and Health Observatory has highlighted the need for further research on ethnic inequalities in access to, experiences of, and outcomes of digital healthcare.^22^

While the DISC study focused on the lived experiences of individuals of a South Asian background, a number of the recommendations developed through our research and workshop align with those from the NHS. This includes the need to work with minority ethnic communities to shape and deliver more equitable services and to undertake thorough evaluations of digital health technologies funded by the NHS.^23^ Studies have also highlighted the importance of hands-on approaches to address disparities in cardiometabolic disease management, including screening patients for digital access, skills and preferences, providing equipment, and working with community organisations.^24^ Following the decommissioning of the NHS Apps Library (a collection of health-related online tools and apps assessed against a range of NHS standards) in 2021, the Organisation for the Review of Care and Health Apps (ORCHA) has launched a Digital Health Formulary for professionals and Health App Library for the public.^25^ Although this is only available at some NHS Trusts, there is a continued need for such tools to be made available to benefit all. While communications to trust leaders have highlighted the need to improve data collection,^26^ our recommendations include specific measures to address this, such as by supporting training for front-line staff.

## Conclusion

Our recommendations highlight specific actions that can be taken at the community, provider, developer and policy level to address digital health inequalities. An integrated approach by combining experiences and efforts from these different groups of stakeholders can ensure that digital health solutions reach the population in need and support healthcare professionals in a meaningful manner.

## Supplemental Material

sj-docx-1-dhj-10.1177_20552076241272600 - Supplemental material for Recommendations for improving accessibility of digital health interventions for cardiometabolic disease for ethnically diverse populations Supplemental material, sj-docx-1-dhj-10.1177_20552076241272600 for Recommendations for improving accessibility of digital health interventions for cardiometabolic disease for ethnically diverse populations by Mel Ramasawmy, Nushrat Khan, David Sunkersing, Madiha Sajid, Shivali H Modha, Manoj Mistry, Wasim Hanif, Fiona Stevenson, Kiran Patel, Kamlesh Khunti, Paramjit Gill, Lydia Poole and Amitava Banerjee in DIGITAL HEALTH
