# Supplementary material for: Recommendations for improving accessibility of digital health interventions for cardiometabolic disease for ethnically diverse populations
Source: Digit Health. 2025 Jan 28;11:20552076241272600. doi: 10.1177/20552076241272600 (PMC11773512; doi:10.1177/20552076241272600)
Supplement: sj-docx-1-dhj-10.1177_20552076241272600 - Supplemental material for Recommendations for improving accessibility of digital health interventions for cardiometabolic disease for ethnically diverse populations [file sj-docx-1-dhj-10.1177_20552076241272600.docx]

# Supplementary 1: Full list of recommendations

## What actions can we take to reduce barriers to, access to, and benefits from, digital health for cardiometabolic disease?

1. Involve patients from app design to implementation.
2. Engage communities to develop trust in digital health.
3. Support training to improve digital skills, such as via community groups or ‘digital occupational therapists’.
4. Research what the public are interested in to ensure DHIs meet their needs.
5. Consider how infrastructure around research and design can make it easier to be inclusive
6. Guidance on how to adapt DHIs to different communities.
7. Programs to make digital devices available to people who need it - such as recycled mobile phones.

Example suggestions from the Menti:

*“Prescribe old recycled phones and provide digital tech training in house or online via you tube using trusted personalities”*

*“Maybe more help and encouragement to be given to people. Community groups for people to attend and to learn”*

*“How do we develop trust – need to engage with populations”*

*“Fear use of digital meant be abandoned by health care team”*

*“Ensure that the tool brings hope to the user – don’t just make it all about risk. Involve users in the design from the outset and test it early on. Provide clear instructions. Consider free web apps”*

## How can we embed learning about inclusive design and patient testing in early development of digital health tools for cardiometabolic disease?

1. Involve target users in the conception, design and testing of DHIs, and recognize this input through compensation for their time.
2. Produce guidance on improving accessibility for different groups in the design process and the end product.
3. Share learning between projects that have successfully designed and implemented DHIs.

Example suggestions from the Menti:

*“DHIs need to be trialed by the target audience in order to get relevant feedback”*

*“Bring all funders together for a workshop on it”*

*“Learning from other successful health programme apps implemented”*

*“Ensure that those who do it well publish on how to do it by way of influencing the funders”*

## How can regulation and evaluation improve how inclusivity is considered in digital health tools for heart disease and diabetes?

1. Develop frameworks for evaluation and that answer not just if it works, but if it works for all in the long-term.
2. Evidence based standards for DHIs for accreditation for NHS use, that share examples and case studies to support evaluation.
3. Clear leadership, governance and accountability, including ensuring those at all levels understand ‘what good looks like’, and the consequences of failing to meet standards.

Example suggestions from the Menti:

*“1. Having governance structure 2. Lines of accountability 3. System leadership”*

*“Standards – need to be improved and have an evidence base. Regulation needs to ensure safety. Give examples/case studies for best practice. Repercussions for not following. Education”*

*“This can be improved through choosing method i.e. DHIs, analysing your data, how the product works, further health economic studies, before & after study/designing your own evaluation using results”*

## How do we improve data quality and access to understand and reduce inequalities related to digital health and cardiometabolic disease?

1. Ensure patients and service users understand the benefits, risks, and limitations of data collection, as well as their rights, in order to build trust.
2. Training and support for staff to help collect potentially sensitive data.
3. Develop meaningful categories for data that address health inequalities, and review these regularly to ensure they are appropriate.

Example suggestions from the Menti:

*“Trusted bodies need to be in place to make sure the data is gathered in a sensitive manner. There needs to be transparency in communicating what the data is used for”*

*“Data quality needs to improve, in order to get good results”*
